# The Collective Investigation of Diphtheria as Conducted by the Therapeutic Gazette, Detroit, Michigan, with Editorial Summary

**Published:** 1883-11

**Authors:** 


**The Collective Investigation of Diphtheria as Conducted by the Therapeutic Gazette, Detroit, Michigan, with Editorial Summary.** By J. J. Mulheron, M. D. Detroit, Michigan: Geo. S. Davis, Medical Publisher, 1883.

This work aims to furnish the profession with the views and opinions and experience of a large number of medical men, obtained in answer to certain questions propounded to them in regard to diphtheria, its nature, treatment and prophylaxis. We endorse the methods taken by the publisher in securing the opinions of a large number of practitioners, and embodying them in a book, which he now introduces to the public, the contents of which contain valuable data and statement in regard to this very serious disease, which will be of service to the profession. We notice that Dr. Gaillard, in his journal, criticizes the work and the methods taken to procure the facts therein set forth. We cannot endorse his criticism. We are in sympathy with this or any other like movement which disseminates information in regard to any of the zymotic diseases, which prevail so largely in this country.

